# Mechanisms of electrical vasoconstriction

**DOI:** 10.1186/s12984-018-0390-y

**Published:** 2018-05-29

**Authors:** Mark Brinton, Yossi Mandel, Ira Schachar, Daniel Palanker

**Affiliations:** 1 Department of Bioengineering, University of Utah, 20 S. 2030 E., Salt Lake City, UT 84112 USA; 2 Faculty of Life Sciences, Bar Ilan University, 5290002 Ramat-Gan, Israel; 3 Department of Ophthalmology, Stanford University, 2452 Watson Court Palo Alto, Stanford, CA 94303 USA; 4 Hansen Experimental Physics Laboratory, Stanford University, 452 Lomita Mall, Stanford, CA 94305 USA

**Keywords:** Electrical stimulation, Electroceuticals, Vasoconstriction

## Abstract

**Background:**

Electrical vasoconstriction is a promising approach to control blood pressure or restrict bleeding in non-compressible wounds. We explore the neural and non-neural pathways of electrical vasoconstriction in-vivo*.*

**Methods:**

Charge-balanced, asymmetric pulses were delivered through a pair of metal disc electrodes. Vasoconstriction was assessed by measuring the diameter of rat saphenous vessels stimulated with low-voltage (20 V, 1 ms) and high-voltage (150 V, 10 μs) stimuli at 10 Hz for 5 min. Activation pathways were explored by topical application of a specific neural agonist (phenylephrine, alpha-1 receptor), a non-specific agonist (KCl) and neural inhibitors (phenoxybenzamine, 25 mg/ml; guanethidine, 1 mg/ml). Acute tissue damage was assessed with a membrane permeability (live-dead) fluorescent assay. The Joule heating in tissue was estimated using COMSOL Multiphysics modeling.

**Results:**

During stimulation, arteries constricted to 41 ± 8% and 37 ± 6% of their pre-stimulus diameter with low- and high-voltage stimuli, while veins constricted to 80 ± 18% and 40 ± 11%, respectively. In arteries, despite similar extent of constriction, the recovery time was very different: about 30 s for low-voltage and 10 min for high-voltage stimuli. Neural inhibitors significantly reduced low-voltage arterial constriction, but did not affect high-voltage arterial or venous constriction, indicating that high-voltage stimuli activate non-neural vasoconstriction pathways. Adrenergic pathways predominantly controlled low-voltage arterial but not venous constriction, which may involve a purinergic pathway. Viability staining confirmed that stimuli were below the electroporation threshold. Modeling indicates that heating of the blood vessels during stimulation (< 0.2 °C) is too low to cause vasoconstriction.

**Conclusions:**

We demonstrate that low-voltage stimuli induce reversible vasoconstriction through neural pathways, while high-voltage stimuli activate non-neural pathways, likely in addition to neural stimulation. Different stimuli providing precise control over the extent of arterial and venous constriction as well as relaxation rate could be used to control bleeding, perfusion or blood pressure.

## Background

For decades, electrical stimulation of cardiac striated muscle has been successfully utilized in pacemakers and defibrillators. Recently, electrical control of vascular smooth muscle has been proposed to treat bleeding in non-compressible wounds [1–3]. Understanding the vasoconstriction pathways activated by electrical stimuli will help create safe and effective devices for electrical control of blood vessels.

Constriction of blood vessel involves both neural and non-neural pathways. Non-neural vasoconstriction mechanisms include mechanical stretching (myogenic) [4, 5], release of endothelin-1 [6, 7] and uridine adenosine tetraphosphate [8] from endothelial cells, circulating hormones (i.e. angiotensin II) [9, 10], and damaged platelets [11]. The dominant neural mechanisms include a fast ionotropic (P2X receptor) pathway activated by adenosine triphosphate (ATP) [12–15]; slower metabotropic (alpha-1 and -2 adrenoreceptor) pathways with the release of norepinephrine [15–21]; and the release of neuropeptide Y, which potentiates constriction from norepinephrine and ATP [22–25].

Electrical stimulation of blood vessels has been used to study the neural pathways in constriction [15–18, 26–29] and dilation [30, 31], including identification of norepinephrine and ATP involvement by in-vitro stimulation of the rat saphenous artery [13]. Direct electric current induces vasoconstriction and thrombosis [32–34] but also causes tissue damage. In-vitro studies have demonstrated both neural and non-neural electrically induced vasoconstriction using a variety of vessel types (pulmonary, somatic and umbilical); however, they used direct or sinusoidal alternating current, which can damage tissue, and could not directly compare the arterial and venous responses to the same stimulation because the vessels were harvested from different locations [28, 29].

Based on our previous studies of vasoconstriction thresholds as a function of pulse duration, frequency, and amplitude [1, 2], we hypothesized that two distinct stimuli (20 V, 1 ms and 150 V, 10 μs pulses at 10 Hz) could constrict the rat saphenous artery to a similar extent. Using these stimuli, we sought to compare the extent and recovery time of arterial and venous constriction in-vivo, describe the underlying pathways, and determine whether the stimulation required for vasoconstriction damages the rat saphenous vessels.

## Methods

### Animals

Male, Long Evans rats (Charles River), aged 50–60 days, with average weight of 309 g (range: 220-380 g) were used in this study with approval by the Stanford Administrative Panel on Laboratory Animal Care. Fourteen animals were used to confirm the maximum constriction using the proposed electrical parameters. In the vasoconstriction and neural inhibition study 7 animals were used for control, 6 for phenoxybenzamine and 5 for guanethidine blockade. Additional five animals were used to assess vessel damage with the live-dead assay. Before surgery, animals were anesthetized using ketamine HCl (75 mg/kg) and xylazine (5 mg/kg), with an additional half dose given every 45 min thereafter.

For surgery, the animal was placed in the supine position and the rectal temperature was kept at 37 ± 1 °C. The saphenous artery and vein were exposed by removing the skin. Hartman’s Lactated Ringer solution (~ 37 °C) dripped at about 1 Hz onto the surgical site during the surgery and stimulation.

### Neural inhibitors

Neural inhibitors were applied topically once the skin was removed and vessels exposed. Phenoxybenzamine HCl (Sigma Aldrich) was dissolved in DMSO (25 mg/ml, Sigma Aldrich). Guanethidine monosulfate (MyBioSource) was first dissolved in de-ionized water (10 mg/ml) and then diluted with DMSO to 1 mg/ml. Control animals (*n* = 7) received DMSO without inhibitors. The solutions were applied liberally (~ 200 μl) to the exposed vessels and covered with a thin piece of plastic to prevent desiccation (*n* = 6 for phenoxybenzamine and *n* = 5 for guanethidine). Fresh solution was added (~ 200 μl) about every 5 min for a total of 25 min. The superficial fascia was removed to improve visualization of the vessel diameters prior to electrical stimulation. Following electrical stimulation, some animals from each group—control (DMSO), phenoxybenzamine and guanethidine—received about 200 μl of potassium chloride (25 mg/ml, Sigma Aldrich; *n* = 5) and, after several minutes of washout, one drop of phenylephrine HCl (25 mg/ml, Akorn; n = 5 for the Guanethidine block and *n* = 7 for the phenoxybenzamine block) as positive controls of vasoconstriction. Potassium chloride acts directly to depolarize nerves and smooth muscle while phenylephrine selectively activates the alpha-1 adrenergic receptors on smooth muscle cells. Because maximal constriction occurred within several seconds, only the maximal constriction from phenylephrine and potassium chloride are reported.

### Vessel stimulation and data collection

**Fig. 1:**
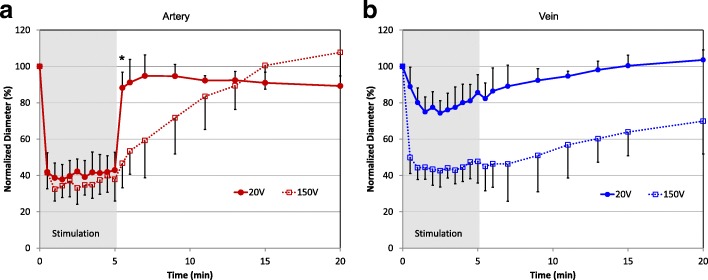
Arterial and venous constriction in response to 10 Hz stimulation at 20 V, 1 ms and 150 V, 10 μs pulses. **a** Both stimuli constrict arteries to similar extent, but arteries dilate faster after low-voltage stimulation (*p < 0.01, F(3,34) = 23.24; one-way ANOVA with Tukey-Kramer multi-comparison test). **b** Veins constrict similarly to arteries at high voltage, but significantly less at low-voltage stimulation (p < 0.01, F(3,34) = 27.31; one-way ANOVA with Tukey-Kramer multi-comparison test on the average vessel diameter during stimulation); *N* = 7 for 20 V and *N* = 12 for 150 V stimuli

Electrical stimulation and video monitoring of the vessels’ width were performed as previously described [2]. Briefly, stainless steel disc electrodes, 1.6 mm diameter, were placed 3.5 mm apart, with the saphenous vessels between them. An anodic square pulse from a customized pulse generator was delivered through an 11 μF capacitor to the electrodes to assure charge balance. Electrical parameters were selected based on previous studies [1, 2], which demonstrated that 150 V, 10 μs stimulation at 1 Hz induced a maximum constriction (about 30% of the original diameter), and 20 V, 1 ms stimulation at 1 Hz produced constriction to 40–45% of the original diameter. Since constriction also increased with pulse frequency, we hypothesized that 20 V, 1 ms (referred to as low-voltage) and 150 V, 10 μs (referred to as high-voltage) stimulation, pulsed at 10 Hz, would both reach the state of maximum constriction in the arteries. We first tested these stimulation parameters in 14 animals (7 animals with both 20 V and 150 V, 5 with only 150 V and 2 with only 20 V stimuli) without pharmacological treatment (Fig. 1). When multiple stimulations were delivered on the same animal, the second occurred at least 15 min later and about 1 cm proximal to first stimulation. Stimulations lasted for 5 min, and the waveforms were monitored using an oscilloscope (Tektronix, TDS 210). The low-voltage stimulus delivered 12.5 mA (250 μJ/pulse) and the high-voltage stimulus 120 mA (180 μJ/pulse), as measured with a 100 Ω series resistor. The inner diameter of blood vessels was measured in ImageJ (NIH) from video captured with a digital camera (Sentech Inc., TC202USB-A).

Data were normalized by the vessel diameter prior to stimulation and presented as mean ± stdev. Statistical significance (*p* < 0.01) was determined using one-way ANOVA, and, where appropriate, the post hoc Tukey-Kramer test to determine statistical significance between study groups. To compare vessel constriction between study groups, the vessel diameters were averaged over the 5-min stimulation period.

### Viability staining and vessel damage

To determine whether the electrical stimulation damaged cell membranes, the saphenous vessels were surgically exposed, as described above, and the femoral artery of anesthetized rats was cannulated with a micro-renethane catheter (Braintree Scientific, MRE-025) [35]. The catheter was advanced to the sapheneous branch, at which time the animal was euthanized. Immediately, the distal end of the saphenous vessel was cut and the vessel flushed with Ringers solution. A viability/cytotoxicity assay (BIOTUM, 30002-T) was pumped through the vessel at a rate of 0.02 ml/min (New Era Pump Systems, NE-300). While the staining solution flowed through the vessel, electrical pulses (20 V, 1 ms; 150 V, 10 μs; and 300 V, 10 μs) were applied at 10 Hz for 2 min to the exposed vessel in ascending order of voltage, moving the electrodes by 5 mm for each voltage setting, along the saphenous vessels. Stimulation at 300 V, 10 μs was included as a positive control to validate the viability/cytotoxicity assay. After the last stimulation, the assay continued to flow through the vessel for another 15 min. The vessel was then again flushed with Ringers solution, excised and mounted on a glass slide for imaging. The cytotoxicity component of the assay (ethidium homodimer III) only enters the cells with damaged membranes to label the nuclei red, while the viability component (calcein AM) crosses uncompromised cell membranes to label the entire viable cell green. The number of damaged cells (red) for each case were counted using ImageJ and normalized by the control.

### Multiphysics modeling

Based on the electric current measured in-vivo, we modeled the electric field and tissue heating using COMSOL Multiphysics. The model assumed symmetry with respect to the plane passing through the middle of the disk electrodes to reduce the modeling volume to 1.5 × 0.5 × 0.7 cm. The tissue was assumed to have electrical and thermal properties of a muscle, and a 0.1 cm thick saline layer covered the 0.6 cm thick muscle layer. Blood vessels in the muscle layer were modeled as 0.6 cm long cylinders of blood. Because constricted vessel diameters (especially the vein) depend on the electrical stimulus applied, we modeled the vessel diameters and flow rates according to our previous work (Table 1) [2]. The vessels passed between two 0.16 cm diameter electrodes, separated by 0.35 cm, center-to-center. Electrodes were placed in direct contact with the muscle tissue, covered with saline.

**Table 1:** In-vivo vessel diameters and flow rates used in the COMSOL modeling

Applied Stimulus	Artery	Vein
	*Constricted Diameter (μm)*	
20 V, 1 ms	200	430
150 V, 10 μs	160	180
	*Flow Rate* [2] *(m/s)*	
20 V, 1 ms	0.15	0.12
150 V, 10 μs	0.09	0.07

To model the electric field in the tissue, we applied voltage pulses and chose tissue conductivity so that the total injected current matched the current measured in-vivo (12.5 mA and 120 mA for the 20 V and 150 V stimuli). The in-vivo currents delivered through the electrodes were measured using a 100-Ohm resistor in series with the electrode and tissue. The current was measured at pulse onset, before the capacitive interface had charged.

The electrode surfaces were defined as equipotential, and all other model boundaries were insulating. To calculate the average Joule heating, the power density from one electrical pulse was multiplied by the duty cycle of the pulsed stimulus (0.01 for 1 ms pulses and 0.0001 for 10 μs pulses). To account for the recharge phase of the pulse, we took the conservative approach and doubled this average power density. In muscle, a perfusion term was included according the bioheat equation [36]. Blood flow rates through the vessels were estimated using the in-vivo measured diameters and published bleeding rates of electrically constricted saphenous vessels in rats of similar weight and electrical parameters (Table 1) [2]. Blood flowed into the model at 37 °C from the rear in both vessels, a simplification justified by the minimal blood heating due to high flow rates. The boundaries of the modeled volume were set to 37 °C to reflect body temperature and the drip of the warm saline onto the vessels. The muscle and saline boundaries in the plane of symmetry were insulating. The electrical and thermal material properties are detailed in Table 2. The tissue temperature reached a steady state within about 25 s of stimulation. For comparison, the tissue heating without blood flow through the vessels was also calculated.

**Table 2:** Electrical and thermal parameters for COMSOL modeling

	Muscle	Blood	Saline
Electrical Conductivity (S/m)	0.376 (20 V)[51] 0.475(150 V)[51]	0.76 [52, 53]	1.0
Thermal Conductivity (W/m∙K)	0.52 [53]	0.5 [54]	0.59
Specific Heat Capacity (J/kg∙K)	3550 [54]	3840 [54]	4173 [55]
Density (kg/m^3^)	1041[53]	1055[56]	1000[56]
Perfusion Parameter (s^− 1^)	0.00067 [53]	NA	NA

## Results

### Electrical stimulation of blood vessels

Upon electrical stimulation, arteries constricted to 41 ± 8% and 37 ± 6% of the initial vessel diameter with 20 V, 1 ms and 150 V, 10 μs pulses repeated at 10 Hz (Fig. 1(a)). While arteries constricted to a similar extent with both stimuli, vessels treated with low-voltage dilated back to 90% of the initial diameter within 30 s, while vessels treated with high-voltage stimulation recovered after 10 min.

With high-voltage stimulation, veins constricted to a similar extent as arteries (40 ± 11% of the initial vessel diameter), but to only 80 ± 18% of the initial diameter with low-voltage stimuli (Fig. 1(b), *p* < 0.01). Veins also recovered slower than arteries—10 min with low-voltage and 15 min with high-voltage stimuli.

### Assessment of tissue damage by electrical stimulation

**Fig. 2:**
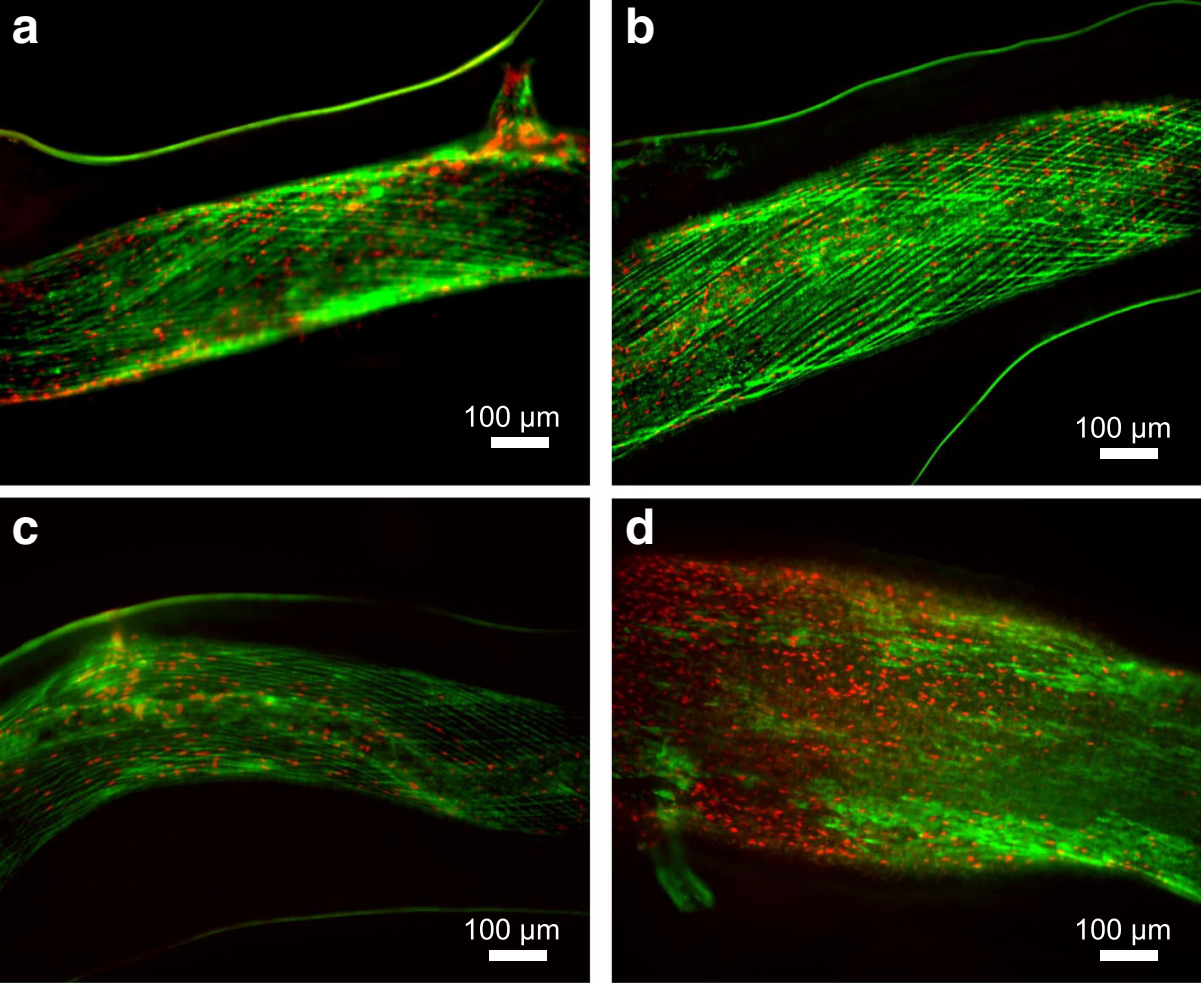
Cell viability assay of the stimulated artery. Green color indicates intact cells, while red shows cells with compromised cell membrane. **a** Control with no stimulation (*N* = 5); **b** 20 V, 1 ms pulses (N = 5); **c** 150 V, 10 μs pulses (N = 5); or **d** 300 V, 10 μs pulses (*N* = 4). All treatments were applied at 10 Hz for 2 min. The number of compromised cells was normalized by the control. Vessels stimulated by 20 V and 150 V pulses exhibited damage similar to the control (1.1 ± 0.8, 1.2 ± 0.3, and 1.0 ± 0.6 a.u., respectively), while the 300 V stimulation damaged 4.7-fold more cells (4.7 ± 1.6 a.u.; *p* < 0.05 and F(3,15) = 18.15, using one-way ANOVA and Tukey-Kramer multi-comparison tests)

To evaluate tissue damage by electrical stimulation (electroporation), we applied a fluorescent cell viability assay to stimulated blood vessels: cells with permeabilized membranes fluoresce in red, while intact cells are stained with green. As shown in Fig. 2, a limited number of damaged cells (red) can be seen in the vessel walls of the control (no stimulation), as well as in the 20 V and 150 V samples. However, with the 300 V stimuli (10 μs pulses at 10 Hz), the number of damaged cells increased 4.7-fold (*p* < 0.05) and the arterial wall dilated beyond its non-stimulated diameter.

### Tissue heating by electrical stimulation

**Fig. 3:**
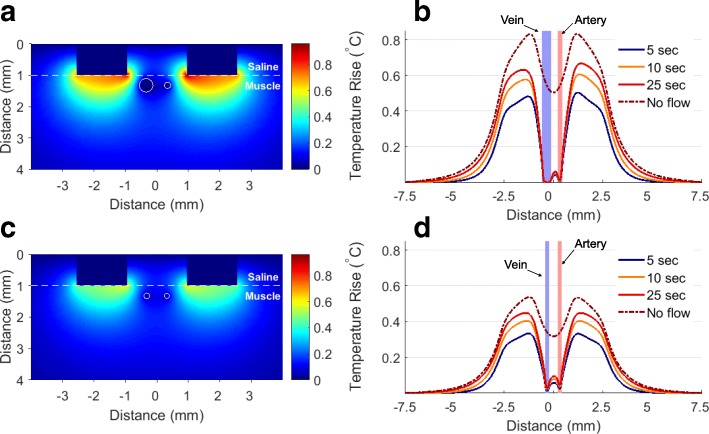
Temperature rise from **a** 20 V, 1 ms and **c** 150 V, 10 μs stimulations, pulsed at 10 Hz. The temperature rise along the line passing through the center of blood vessels for **b** 20 V and **d** 150 V stimulation

To assess the extent of tissue heating during stimulation, we modeled the system using COMSOL Multiphysics. At steady-state, about 25 s of stimulation, the temperature on electrode surface increases by about 0.96 °C with 20 V and by 0.61 °C with 150 V stimuli (Fig. 3). Near blood vessels, the steady-state temperature rise was only 0.2 °C and 0.15 °C from the 20 V and 150 V stimuli, respectively. As a worst-case scenario, we modeled tissue heating without blood flow in the vessels, which yielded temperature rise of 0.6 °C and 0.35 °C at the vessel walls. Both temperatures are well within the range of physiological variations, and less than the temperature change from each drip of warmed saline on the vessel surface.

### Neural pathways

**Fig. 4:**
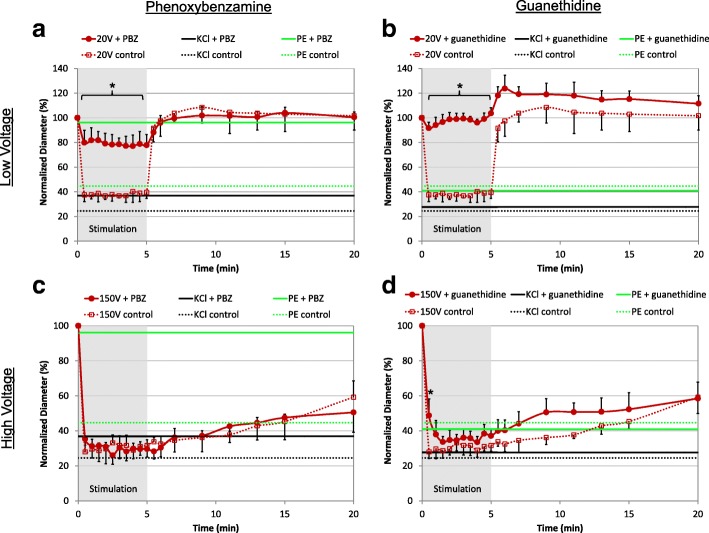
Neural inhibition of arterial vasoconstriction. **a** Phenoxybenzamine (PBZ) completely blocked constriction induced by phenylephrine (PE, *p* < 0.01), slightly inhibited constriction induced by potassium chloride (KCl, *p* = 0.02), and significantly decreased constriction induced by 20 V stimulation (*p < 0.01, F(11,58) = 98.75; one-way ANOVA and Tukey-Kramer multi-comparison test). **b** Guanethidine had no effect on vasoconstriction induced by PE or by KCl, but it blocked the 20 V-induced constriction (*p < 0.01). **c** PBZ did not inhibit constriction induced by 150 V stimulation, demonstrating that vasoconstriction by high-voltage bypassed the adrenergic neural pathway. **d** On average, guanethidine failed to block constriction induced by 150 V stimuli. However, the onset of constriction was slowed down (*p < 0.01; one-way ANOVA, F(1,9) = 25.14). Unless specified, significance was determined using one-way ANOVA and Tukey-Kramer multi-comparison test, F(11,58) = 98.75; N = 5 for guanethidine, *N* = 6 for PBZ and N = 7 control. Horizontal lines indicate the maximum constriction achieved with phenylephrine (green) and potassium chloride (black)

To determine whether neural and non-neural pathways were involved in electrically-induced vasoconstriction, we applied a selective neural agonist (phenylephrine), neural inhibitors (phenoxybenzamine and guanethidine), and a non-specific depolarizing agent (potassium chloride). Phenylephrine is a synthetic analog of norepinephrine that binds and activates the alpha-1 adrenergic receptor. Phenoxybenzamine (PBZ) prevents binding to the alpha-1 and alpha-2 adrenergic receptors on smooth muscle cells. Guanethidine prevents the release of adrenergic (norepinephrine) and purinergic (adenosine triphosphate) neurotransmitters from sympathetic nerves. Potassium chloride (KCl) depolarizes both nerves and smooth muscle cells directly. Since maximal constriction occurred immediately following application, we plot these levels of constrictions as horizontal lines in Fig. 4, for comparison.

#### Neural inhibition: Arteries

As expected, phenylephrine induced a strong arterial constriction to 45 ± 16% of the initial vessel diameter (Fig. 4, green dash). Pretreatment with PBZ completely blocked arterial constriction with phenylephrine—the vessel constricted to only 96 ± 5% of the initial diameter (*p* < 0.01; Fig. 4(a) and Fig. 4(c), green solid). Potassium Chloride (KCl) produced the largest arterial constriction, down to 25 ± 2% of the initial diameter (Fig. 4, black dash). PBZ slightly reduced the constrictive effect of KCl, presumably by preventing the endogenous norepinephrine, released from depolarized nerves, from binding to the adrenergic receptors (alpha-1 and alpha-2) on the smooth muscle cells; however, KCl can also act on smooth muscle directly so the arterial diameter increased only slightly from 25 ± 2% to 37 ± 4% of the initial (*p* < 0.01; Fig. 4(a) and Fig. 4(c), black). As expected, Guanethidine failed to inhibit constriction induced by phenylephrine because phenylephrine acts downstream of the guanethidine blockade: 45 ± 16% (without guanethidine) compared with 40 ± 3% (with guanethidine) of the initial arterial diameter (Fig. 4(b) and Fig. 4(d), green). Guanethidine also had a limited effect on KCl, which can act directly on smooth muscle cells and constricted the vessel to 25 ± 2% of the initial vessel diameter without guanethidine, compared with 30 ± 4% with guanethidine (Fig. 4(b) and Fig. 4(d), black).

Pretreatment with PBZ reduced arterial constriction with low-voltage (20 V) stimuli and increased the vessel diameter from 38 ± 4% (without PBZ) to 79 ± 7% (with PBZ) of the original (*p* < 0.01; Fig. 4(a), red). Pretreatment with guanethidine completely eliminated arterial constriction with low-voltage (20 V) stimulation—the stimulated vessel diameter increased from 38 ± 4% (without guanethidine) to 98 ± 2% (with guanethidine) of the pre-stimulus value (*p* < 0.01; Fig. 4(b), red). Guanethidine pretreatment also revealed a post-stimulus dilation up to 127% of the initial diameter, which trends back to normal over 15 min.

With high-voltage stimulation, PBZ and guanethidine failed to block arterial vasoconstriction: the vessel diameters were 30 ± 3% of the initial without PBZ or guanethidine, 30 ± 6% with PBZ, and 37 ± 2% with guanethidine (Fig. 4(c) and Fig. 4(d), red). However, in the presence of guanethidine, arterial constriction occurred slower: at 30 s the artery constricted to 28 ± 4% of the initial diameter in the control but just 51 ± 11% of the initial diameter with guanethidine (*p* < 0.01; Fig. 4(d), red).

#### Neural inhibition: Veins

**Fig. 5:**
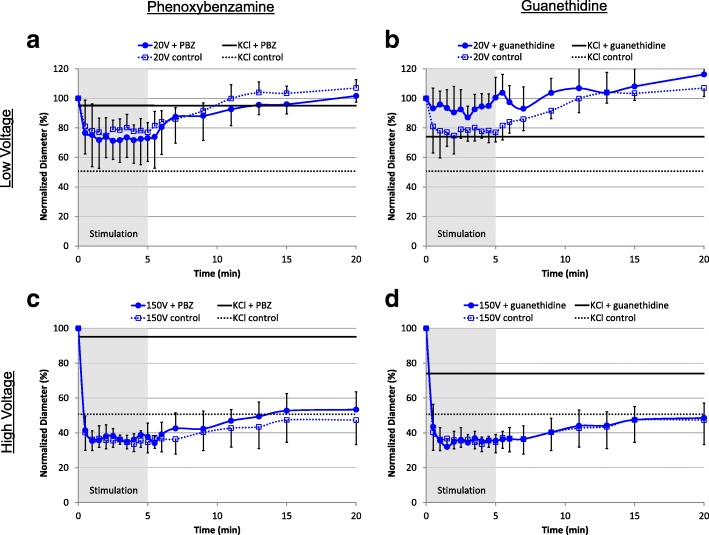
Neural inhibition of venous vasoconstriction. **a** PBZ did not block venous constriction induced by 20 V stimulation, but it reduced constriction induced by KCl (p = 0.02). **b** Although guanethidine reduced constriction induced by 20 V stimulation and by KCl, the differences were not significant. **c** PBZ did not affect vasoconstriction induced by 150 V stimulation. **d** Guanethidine did not affect constriction induced by 150 V stimulation. Horizontal lines indicate the maximum constriction achieved with potassium chloride (black). Statistical comparisons were performed using the one-way ANOVA and the Tukey-Kramer multi-comparison test; N = 5 for guanethidine, N = 6 for PBZ and N = 7 for control

The neural agonists and antagonists affected veins quite differently. Phenylephrine (PE) failed to elicit a venous contraction and was not affected by the neural inhibitors: the vein diameters were 97 ± 13% (PE alone), 102 ± 7% (with PBZ) and 92 ± 10% (with guanethidine). PBZ reduced venous constriction with KCl and increased the vessel diameter from 51 ± 21% of the initial to 95 ± 6% with PBZ (*p* = 0.02). The increase to 74 ± 19% with guanethidine was not significant (Fig. 5, black).

PBZ did not affect the venous response to low-voltage stimulation (78 ± 9% of the pre-stimulus diameter compared with 73 ± 15% with PBZ), while guanethidine reduced constriction, but not significantly (94 ± 10% of the pre-stimulus diameter) (Fig. 5(a) and Fig. 5(b), blue). Neither PBZ nor guanethidine affected venous constriction by high-voltage stimulation: vein diameters were 36 ± 4% of the pre-stimulus diameter (control), compared with 37 ± 4% (PBZ) and 36 ± 5% (guanethidine) (Fig. 5(c) and Fig. 5(d), blue).

## Discussion

### Electrical stimulation of blood vessels

We found that both, high- and low-voltage stimuli constrict saphenous arteries to a similar extent in-vivo, but low-voltage engages a neural pathway that recovers quickly (within 30 s), while high-voltage activates a non-neural pathway that recovers slowly (over several minutes). We also show that high-voltage stimulation constricts veins as much as arteries, but low-voltage constricts only half that amount. These observations suggest that different vasoconstriction pathways could be activated by electrical stimulation.

### Electrical stimulation below damage threshold

Strong electric field can permeabilize and damage cell membranes; however, our cellular viability assay showed no damage to arteries with the 20 and 150 V stimuli. Extremely high electric field (tens of kV/cm) with pulse durations shorter than the typical cell polarization time (~ 50 ns) can selectively polarize intracellular organelles [37]. While damage of intracellular organelles would not be detected with our viability assay, it is unlikely that our stimulus induced any direct action on them because electric field in our case is about 2 orders of magnitude lower than that required for activation of intracellular organelles. Extremely high electric fields can also activate platelets in the blood, which may constrict vessels by releasing thromboxane or serotonin [11, 38, 39], but this pathway is also unlikely due to the substantially lower electric fields in our study.

With 150 V, 0.01 ms stimuli, charge density at the electrode surface (60μC/cm^2^) is close to the capacitive coupling limit for stainless steel-electrolyte interface (40-50μC/cm^2^), and may be delivered without electrolysis due to surface roughness. For the 20 V, 1 ms stimulus, charge density (625μC/cm^2^) exceeds the capacitive coupling limit, so the current was sustained via electrolysis of water [40]. However, even with electrolysis, it is unlikely that gas byproducts or changes in pH affected vasoconstriction since the electrodes were located several millimeters away from the vessels and warm saline was continuously washing the tissue surface. To avoid hydrolysis in clinical applications, electrodes should have sufficiently high capacitance, such as sputtered iridium oxide films (SIROF), which can safely supply charge densities exceeding 1mC/cm^2^ [40].

### Heating by electrical stimulation

For electric field modeling, we selected the muscle conductivity, so that the total current matched the in-vivo measured current (12.5 mA or 120 mA for the 20 or 150 V electrodes). This approach resulted in a slightly lower value of muscle conductivity for the 20 V stimulus (likely due to gas formation at the electrode-electrolyte interface). Our thermal modeling demonstrated a temperature rise below 1 °C on electrodes, and only 0.15–0.2 °C on the vessel walls, even without considering cooling from convection at the exposed saline surface. Such a minimal heating is very unlikely to induce vasoconstriction since temperature pulsation by a few degrees from a drip of warm saline (37 °C) did not affect the vessel diameter. The vessel heating is similar to our previous reported values (about 2.5 °C with 150 V, 100 μs pulses at 10 Hz and 0.02 °C with 80 V, 1 μs pulses at 10 Hz) [1]; however, our current model predicts even less heating with 150 V pulses because of about 10-fold less charge per pulse and luminal blood flow based on in-vivo measurements. While variations in blood flow affect the modeling results, even without blood flow, the vessels will heat no more than 0.6 and 0.35 °C for 20 V and 150 V stimuli—again less temperature variation than that produced by the dripping 37 °C saline.

### Neural pathways

The thermal modeling and cell viability assay suggest that vasoconstriction was not induced by electroporation or vessel heating. To understand the mechanisms of electrical vasoconstriction we applied the pharmacological inhibitors PBZ and guanethidine. PBZ partially blocks neuro-mediated constriction by preventing the neurotransmitter (norepinephrine) from binding to alpha-1 and alpha-2 receptors on the smooth muscle cells [13], while guanethidine provides a complete neural block by preventing the release of adrenergic (norepinephrine) and purinergic (adenosine triphosphate) neurotransmitters from sympathetic nerves [15].

### Neural inhibition during low-voltage stimulation

In-vivo*,* low-voltage constriction in arteries was neuro-mediated, with about 65% of the effect due to the adrenergic pathway and additional 30–35% from the purinergic pathway, as evidenced by the partial and complete inhibition with PBZ and guanethidine, respectively. Neural vasoconstriction through adrenergic (dominant) and purinergic pathways was also observed ex-vivo in rat saphenous arteries using similar electrical parameters [13]. Low-voltage stimulation did not depolarize the arterial smooth muscle directly in-vivo (evidenced by complete blockage of vasoconstriction with guanethidine) which confirmed ex-vivo observations [13].

PBZ completely eliminated venous constriction by KCl, while it was only slightly reduced in case of arterial constriction by KCl. This implies that KCl induces venous constriction by depolarizing neurons that release norepinephrine. Because phenylephrine, a pure alpha-1 agonist, did not affect the vein, we conclude that saphenous vein constriction occurs primarily through the alpha-2 receptors, which are activated by norepinephrine, blocked by PBZ and unaffected by phenylephrine. The alpha-2 receptor pathway was also shown to be the dominant venous constriction pathway in dogs [19, 20]. Interestingly, the adrenergic pathway (alpha-1 and -2 receptors) does not appear to be involved in low-voltage venous constriction because pretreatment with PBZ failed to block constriction. Low-voltage venous constriction may involve activation of a purinergic pathway because veins treated with guanethidine constricted less than without purinergic blockage (Fig. 5(b)).

Low-voltage, neural stimulation primarily affects arterial constriction and flow, which could be useful to control hemorrhage [2], blood perfusion or blood pressure in a localized tissue or organ. The neural pathway provides rapid constriction and dilation and can safely constrict vessels for hours [2]. However, chronic stimulation will require electrode materials capable of safely injecting 625μC/cm^2^, such as SIROF or TiN [40, 41].

Arterial dilation following low-voltage stimulation was observed most clearly in guanethidine treated vessels (Fig. 4(b)), and it may be mediated by release of nitric oxide or prostaglandins [42, 43]. Because the dilation presented only when the neurotransmitters were blocked, the dilatory effect appears to be overpowered under normal stimulation conditions (no pharmacological blockade). Further studies could determine whether this effect could be exploited to increase blood flow in tissue with poor circulation.

### Neural inhibition during high-voltage stimulation

In-vivo*,* high-voltage vasoconstriction was not dependent on a neural pathway, since it was not affected by neurotransmitter blockers and confirms previous in-vitro studies showing both arterial and venous constriction in the presence of neural inhibitors [28, 29]. Direct depolarization of smooth muscle with high-voltage stimuli is unlikely because high-voltage constriction persists for several minutes after stimulation, unlike KCl-induced constriction which directly depolarizes smooth muscle and reverses within a minute of rinsing the solution. Furthermore, it has been shown that contractility of smooth muscle decreased rapidly below 165μC/cm^2^ per pulse at 20 Hz [44]. Our high-voltage stimulation generates 8-fold less charge density per pulse (20μC/cm^2^) at the arterial wall with half the pulse frequency (10 Hz), further indicating that a direct effect on smooth muscle is unlikely in our case.

High-voltage electrical vasoconstriction may result from release of endothelin-1 by endothelial cells in the lumen of arteries and veins: endothelin-1 constricts vessels to a similar extent as KCl, and does not readily wash-out (vessels remain constricted for more than 10 min) [6, 7, 45, 46]. Endothelial cells under mechanical stress can also release uridine adensosine tetraphosphate and induce potent vasoconstriction [8]. Since vasoconstriction is localized between the electrodes, circulating agents (such as angiotensin) are unlikely to play a role because they would diffuse downstream rather than constrict the vessel only locally.

For some applications, high-voltage, non-neural vasoconstriction has the advantage of constricting veins nearly as much as arteries. This could help control traumatic bleeding in highly perfused tissue, where the major arterial blood supply may be difficult to locate or reach, or in sacral and pelvic cavities where venous hemorrhage can be significant [47–49].

Since high-voltage stimulation uses 40% less energy per pulse, achieves maximum constriction with 10-fold lower pulse frequency [1], and could be applied intermittently because constriction lasts several minutes, it could enable smaller, more power efficient devices for long lasting vessel control. At 1 Hz, high-voltage delivers 14-fold less power than the low-voltage stimulation.

### Limitations

One limitation of this study is that we have not shown safety for clinically relevant durations of stimulation (i.e. greater than 30 min). However, histological examination of the rat saphenous vessels showed no vessel damage one week after a 60-min-long stimulation with identical electrodes at low voltage (20 V, 1 ms pulses at 10 Hz) [2]. In addition, a previous study demonstrated that the threshold of cellular damage by electroporation does not decrease beyond about 50 pulses, suggesting that longer stimulations should also be safe [50].

The DMSO used in the inhibitor experiments extended the arterial recovery time after high-voltage constriction (comparing Fig. 1(a) and Fig. 4(c)). However, it did not affect the extent of constriction, so comparisons between neural inhibitors and their controls are accurate. Even without DMSO (Fig. 1(b)), the vein did not fully dilate 15 min after high-voltage stimulation, perhaps due to lower blood pressure compared with the artery.

Electrical stimulation capable of inducing vasoconstriction also activates the nearby muscles and sensory nerves. Due to depletion of acetylcholine at the neuromuscular junction, muscle contraction with each stimulus pulse decreased over time, and was almost gone after about a minute. Unwanted activation of the muscle and sensory nerves could be reduced by using sensors to identify and stimulate only near the source of hemorrhage, by using neuromuscular blocking agents available during general anesthesia or by intermixing the vasoconstriction stimuli with high pulse frequency stimuli, capable of blocking the pain or completely exhausting the neuromuscular junction.

## Conclusions

Pulsed electrical stimulation provides a reversible and non-damaging approach to blood vessel control in-vivo. Low-voltage stimuli engage neural vasoconstriction pathways, while high-voltage also activates non-neural pathways to induce maximum arterial constriction. The low- and high-voltage stimuli provide different extent of constriction and rates of dilation, which could be useful in a variety of applications for control of bleeding, perfusion, or blood pressure.

## Abbreviations

KCl: Potassium chloride
PBZ: Phenoxybenzamine
